# Supplementary material for: Sugar-Sweetened Beverage Consumption and Associated Health Risks Awareness Among University Students in Kuwait: A Cross-Sectional Study
Source: Nutrients. 2025 May 12;17(10):1646. doi: 10.3390/nu17101646 (PMC12114537; doi:10.3390/nu17101646)
Supplement: Supplementary file 1 [file nutrients-17-01646-s001.zip › nutrients-3606623-supplementary.pdf]

Table S1 Supplementary: Sugar content of most popular SSBs available in Kuwait.

SSB	Serving Size	Sugar Content g/100 ml*	Sugar (g)/ serving
Coca-Cola	250 ml can	10.6	26.5
Sprite	330 ml can	11.8	38.94
Fanta	330 ml can	14.5	47.85
Dr. Pepper	500 ml bottle	11.6	58
Pepsi	250 ml can	11	27.5
7-Up	125 ml can	11	13.75
Miranda	200 ml can	14	28
RC-Cola	250 ml can	11	27.5
Mountain Dew	300 ml can	12	36
Red Bulls	500 ml bottle	11	55
Power Horse	250 ml can	11	27.5
Monster Energy	300 ml bottle	11	33
Gatorade Sports drink	320 ml can	6	19.2
Lipton Sweet tea	330 ml	5.9	19.47
NAI Moroccan Mint tea	330 ml	5.9	19.47
Epsa Lemon tea	330 ml	8	26.4

*The sugar content values are based on the SSBs' labels provided by the manufacturers.
